# Challenges Faced by Emergency Physicians in China: An Observation From the Perspective of Burnout

**DOI:** 10.3389/fpsyt.2021.766111

**Published:** 2021-11-17

**Authors:** Shijiao Yan, Xin Shen, Rixing Wang, Zhiqian Luo, Xiaotong Han, Yong Gan, Chuanzhu Lv

**Affiliations:** ^1^School of Public Health, Hainan Medical University, Haikou, China; ^2^Key Laboratory of Emergency and Trauma of Ministry of Education, Hainan Medical University, Haikou, China; ^3^Department of Social Medicine and Health Management, School of Public Health, Tongji Medical College, Huazhong University of Science and Technology, Wuhan, China; ^4^Department of Emergency, Hainan Clinical Research Center for Acute and Critical Diseases, The Second Affiliated Hospital of Hainan Medical University, Haikou, China; ^5^Emergency and Trauma College, Hainan Medical University, Haikou, China; ^6^Research Unit of Island Emergency Medicine, Chinese Academy of Medical Sciences (No. 2019RU013), Hainan Medical University, Haikou, China; ^7^Department of Emergency Medicine, Hunan Provincial Key Laboratory of Emergency and Critical Care Metabolomics, Hunan Provincial Institute of Emergency Medicine, Hunan Provincial People's Hospital/The First Affifiliated Hospital, Hunan Normal University, Changsha, China; ^8^Department of Emergency Medicine, Sichuan Provincial People's Hospital, University of Electronic Science and Technology of China, Chengdu, China

**Keywords:** burnout, emergency medicine, emergency physicians, organizational psychology, prevalence

## Abstract

**Background:** Burnout is considered a global problem, particularly in the emergency health sector; however, no large-sample cross-sectional study has assessed the prevalence of burnout among emergency physicians and its associated factors.

**Methods:** A nationally representative cross-sectional survey of 15,243 emergency physicians was conducted in 31 provinces across China between July and September 2019. Multiple linear regression analysis was performed to identify correlates of burnout.

**Results:** The participants' mean scores were 25.8 (SD = 15.9) on the emotional exhaustion (EE) subscale, 8.1 (SD = 7.9) on the depersonalization (DP) subscale, and 26.80 (SD = 12.5) on the personal accomplishment (PA) subscale, indicating a pattern of moderate EE, moderate DP, and high PA. The results of the large-sample survey found that 14.9% of emergency physicians had a high level of burnout in China, with 46.8% scoring high for EE, 24.1% scoring high for DP, and 60.5% having a high risk of low PA. Having poor self-perceived health status and sleep quality, working in developed regions and governmental hospitals, having an intermediate professional title, experiencing depression, performing shift work and experiencing workplace violence made emergency physicians more likely to experience occupational burnout.

**Conclusion:** Positive measures should be taken to reduce the burnout of emergency physicians and improve their work enthusiasm to maintain the quality of emergency medical services.

## Background

Burnout is a psychological syndrome that is a reaction to the long-term, accumulated negative effect of chronic work-related stress (1). It is a syndrome characterized by feelings of overextension and the depletion of resources (emotional exhaustion, EE), negative or callous responses to job responsibilities (depersonalization, DP), and feelings of incompetence and a lack of achievement (decreased personal accomplishment, PA) (2). DP is often referred to a coping strategy, while PA could be a way of burnout addressing. Burnout has been shown to exert adverse effects on organizations (e.g., turnover, higher absenteeism, lack of job commitment, and job dissatisfaction), the mental and physical health of healthcare practitioners, and the quality of healthcare delivery (2–5). In the United States alone, physician burnout has an estimated economic burden of $4.6 billion a year (6). Therefore, a growing number of researchers have studied physician burnout and found that emergency physicians have the highest burnout rate of any physician group (7–10).

Emergency physicians, who often need to provide urgent care and make important decisions that can affect whether a patient recovers or dies (11, 12), face high levels of stress and are more prone to burnout (13, 14). Emergency physicians often need to deal with numerous patients and various diseases during providing medical services; while they also faced staff shortages and the chaotic work environment characterized by unpredictability in the medical setting. Although many studies on burnout among emergency physicians have been conducted in recent years (15, 16), according to a recent systematic review, the samples of these studies are often small and underrepresentative (*N* = 23–315) (17). Specific data in low-income and middle-income countries are particularly scarce (18). In addition, systematic exploration of the potential factors associated with physicians' burnout is not available (17). Therefore, it is urgent to carry out large-sample studies and clarify the influencing factors on the occupational burnout of emergency physicians.

China has the largest number of emergency patients every year around the world, with more than 166.5 million (19), and China is also the country with the most emergency physicians, with nearly 60 thousand, accounting for more than 2% of all doctors in the country (19). Emergency treatment plays an important role in China's medical system, and it has received ample attention from the government (20). However, according to previous studies, emergency physicians in China still face many underlying dilemmas, including the following:

1. Emergency physicians in China are often under great work pressure. Work pressure may have an impact on their daily lives (21), such as reducing their sleep quality (22, 23), and may also have an impact on their mental health (24, 25), such as triggering psychological problems such as depression (26).
2. An excessive work burden can easily cause health damage (22). Many emergency physicians still need to keep working even when they are ill, which further aggravates the decline in emergency physicians' physical health (27), as well as their mental health.
3. There are regional and sociodemographic differences in China's demand for health resources (28, 29). Hospitals in developed areas or government in China are often faced with an excessive demand for health services and need to provide adequate medical services (30), while hospitals in underdeveloped areas and non-governmental hospitals have an insufficient number of patients (31–33). The imbalance between health resources and needs exacerbates the work burden of emergency physicians in hospitals in developed areas and government-run hospitals.
4. Many hospitals in China have not yet established reasonable management systems, and the management of health human resources is not based on scientific evidence (27, 34). For example, many emergency physicians have to engage in shift work, which greatly increases their work burden (35, 36).
5. In recent years, physician-patient conflicts in China have become more frequent (37, 38). As emergency physicians often deal with patients in the acute stage of illness, they are more vulnerable to verbal or physical violence from patients or their relatives (39). Estrangement between physicians and patients reduces physicians' enthusiasm and sense of achievement.

The difficulties faced by emergency physicians in China reflect the contradictions faced by physicians in China and the deficiencies in health and healthy development. However, the potential impact of these challenges on emergency physicians and the mechanism by which these factors may lead to emergency physicians' burnout are unclear. This study aim to investigate these issues to further develop emergency care, maintain the stability of the population of emergency physicians and determine the influencing factors that have not previously been examined. As China is the largest developing country, understanding the prevalence of burnout among emergency physicians in China and its influencing factors can provide a reference for policy makers and researchers in global health care.

## Methods

### Ethics Statement

The study protocol was approved by the Institutional Ethics Board of the Second Affiliated Hospital of Hainan Medical University, Haikou, China (HYLL-2018-035). All individuals provided written informed consent.

### Study Participants and Survey Design

A cross-sectional study was carried out in China from July 2019 to September 2019. A multistage stratified random sampling design was used in this study. First, a total of 31 Chinese provinces were classified as developed, developing, or less-developed regions according to per capital household income in 2018. Second, we selected 10 hospitals randomly from each province. Third, based on the number and scale of the hospitals, 40% of the emergency physicians who had practiced in the emergency department for at least 6 months were randomly selected from each hospital to complete a self-administered questionnaire. The inclusion criteria for study participants were: (1) emergency physicians performing emergency medicine services; (2) Have been working for at least 6 months. Exclusion criteria for the study were: (1) nurses or other medical technical service personnel; (2) Have worked for <6 months. In total, 15,455 emergency physicians were asked to participate in this survey, and 182 physicians did not respond. Additionally, 30 questionnaires were discarded due to missing information. Ultimately, 15,243 eligible questionnaires were used in this analysis, and response rate was 98.6%.

### Instrument and Measurement

The questionnaire was designed based on literature reviews, group discussions, and preliminary interviews. Independent variables were selected based on previous studies and findings, including demographic characteristics, workplace violence, shift work, and depression. Furthermore, a pilot study was conducted in one Wuhan community to improve the quality of the questionnaire. A total of 50 physicians filled out the questionnaire and made sure the questions were understood. The questionnaire consisted of questions on sociodemographic information (e.g., region, age, gender, education level, marital status, and professional title), burnout, depression status and workplace violence.

Burnout was measured with the Maslach Burnout Inventory (MBI-HSS) that consisted of three subscales and 22 items on a six-point Likert scale ranging from 0 (never) to 6 (every day) (40). The three dimensions of EE, DP, and PA were measured in 9, 5, and 8 items, respectively. Higher scores on the EE and DP subscales were positively associated with higher levels of burnout, while the PA subscale score was inversely associated with burnout. In the analysis of the prevalence of burnout, we analyzed the subscales as categorical variables. The cutoff points for different categories (low, moderate, and high) for each subscale were defined in the study according to the MBI-HSS scoring guidelines (41). Emergency physicians were categorized as having a high level of burnout if they scored high on EE and DP and low on PA. In this study, the Cronbach's alpha coefficients for the MBI-HSS total scale and the EE, DP, and PA subscales were 0.86, 0.92, 0.88, and 0.89, respectively, suggesting that the overall measurement was reliable.

The Center for Epidemiological Studies Depression scale (CES-D) was used to assess depression. The scale includes includes 20 items; each item is scored on a four-point scale ranging from 0 (“little or none of the time”) to 3 (“most or almost all of the time”). The items are declarative expressions such as “I feel I can't get out of my gloomy mood even with the help of my family and friends.” “I can't concentrate.” The total score ranges from 0 to 60 points, and the higher the score is, the more severe the depressive symptoms. On the original CES-D scale, a total score of 16 was used to detect the presence of depressive symptoms (42). However, a large number of studies have assessed the diagnostic accuracy of CES-D in detecting depression in the general population and have proposed multiple cutoff points, such as a cutoff point of 18 for elderly people living in residential homes (43) and a cutoff score of 22 for older Chinese individuals (44). A meta-analysis systematically reviewed 28 CES-D studies, including several Chinese studies, and obtained an optimal cutoff point of 20 points (45). As a result, an overall score of 20 or higher was considered an indicator of depressive symptoms in this study, consistent with previous research (46). The CES-D has good reliability and validity, and it has been widely used in the Chinese population. In this study, the Cronbach's alpha coefficient of the scale was 0.90.

Workplace violence was measures by Workplace violence Scale (WVS) was developed by Wang et al. (47), has a good reliability and validity for measuring the incidence of workplace violence when applied to medical staff in China. It includes 5 items measured with a four-point ordinal scale ranging from 0 (never) to 3 (more than 3 times/year). In this study, the Cronbach's alpha for WPV was 0.81.

### Data Collection and Quality Control

Our research includes the following steps: (1) determining research objectives; (2) Define the research type; (3) Determine the research object; (4) Determine sample content and sampling method; (5) Data collection and statistical analysis. A web link to the online questionnaire, which was designed using Questionnaire Star, was disseminated to the participants through WeChat (similar to WhatsApp in Western countries, WeChat is the largest communication platform in China, with over one billion users). To prevent the same participants from repeatedly answering the questionnaire, each device (e.g., smartphone or computer) was eligible to complete the questionnaire only once, and logical checks were concurrently run on the WeChat platform to identify invalid questionnaires. The data were entered into a web-based database by trained investigators to ensure accuracy.

### Statistical Methods

In the descriptive analysis, the mean and standard deviation were calculated for continuous variables, and the quantity and percentage were calculated for categorical data. The dependent variables (EE, DP, PA, and overall burnout) were treated as continuous variables. The ANOVA were used to compare differential factors for burnout. No clustering was observed among the respondents (correlation = 0.03, *P* < 0.001). A multiple linear regression model was performed to identify the correlates of three distinct dimensions of burnout, as identified by EE, DP, and PA subscales. Predictive variables such as age (continuous), gender, marital status, education level, work tenure, average monthly income, contract status, professional level, managerial responsibility, practice setting, and workplace violence were included in the multiple linear regression analysis. All statistical analyses were performed with Statistical Analysis System (SAS) version 9.2 (SAS Institute Inc., Cary, NC, USA). Statistical significance was accepted at the 5% level (*P* < 0.05).

## Results

Table 1 presents the main characteristics of the survey respondents. Among the 15,243 respondents, most were married (83.3%), were men (69.9%) and had a bachelor's degree or above (94.2%). The mean age (SD) was 37.66 (8.1) years. Approximately 65% of the participants had intermediate or senior professional titles. In total, 55.23% of the respondents were in the middle- and lower-income groups, more than half estimated their own health to be at the general level, and ~40% were from developing regions. A minority of the participants (27.2%) participated in regular physical activity (Table 1).

**Table 1:** Statistical description of study samples.

**Variables**	***N* (%)**	**EE scores [M (SD)]**	***P*-value**	**DP scores [M (SD)]**	***P*-value**	**PA scores [M (SD)]**	***P-*value**
Total	15,243 (100.00)	25.78 (15.94)	NA	8.13 (7.85)	NA	26.80 (12.53)	NA
**Gender**							
Male	10,650 (69.87)	25.36 (16.05)	<0.001	8.46 (8.08)	<0.001	26.46 (12.77)	<0.001
Female	4,593 (30.13)	26.76 (15.64)		7.38 (7.23)		27.61 (11.91)	
**Age group, y**							
≤31	4,089 (26.83)	24.96 (15.76)	<0.001	8.31 (7.72)	<0.001	25.45 (12.37)	<0.001
>31 and ≤ 37	4,117 (27.01)	28.04 (15.69)		9.20 (8.22)		26.05 (11.86)	
>37 and ≤ 43	3,291 (21.59)	27.00 (15.85)		8.46 (7.92)		27.05 (12.23)	
>43	3,746 (24.59)	23.13 (16.04)		6.48 (7.21)		28.90 (13.37)	
**Region**							
Developed	6,000 (39.36)	25.65 (15.80)	0.431	8.13 (7.74)	0.991	27.03 (12.28)	<0.001
Middle-developed	4,097 (26.88)	26.05 (15.83)		8.14 (7.88)		27.20 (12.37)	
Less-developed	5,146 (33.76)	25.72 (16.19)		8.12 (7.95)		26.23 (12.91)	
**Education level**							
Associate's degree or vocational diploma^*^	894 (5.86)	20.66 (15.76)	<0.001	5.93 (6.86)	<0.001	26.58 (13.91)	0.507
Bachelor degree	10,293 (67.53)	25.92 (15.94)		8.17 (7.92)		26.88 (12.57)	
Master degree or higher	4,056 (26.61)	26.56 (15.80)		8.52 (7.79)		26.65 (12.10)	
**Marital status**							
Married/widow/divorced	12,691 (83.26)	25.88 (15.94)	0.099	8.06 (7.85)	0.012	26.99 (12.56)	<0.001
Unmarried	2,552 (16.74)	25.31 (15.93)		8.49 (7.84)		25.87 (12.34)	
**Income level**							
High	1,672 (10.97)	21.64 (15.47)	<0.001	6.87 (7.25)	<0.001	28.31 (13.40)	<0.001
Middle	6,746 (44.26)	25.04 (15.37)		7.73 (7.45)		27.17 (12.26)	
Low	6,825 (44.77)	27.53 (16.36)		8.84 (8.30)		26.07 (12.52)	
**Work tenure, y**							
≤3	4,921 (32.28)	24.41 (15.75)	<0.001	7.87 (7.52)	<0.001	26.03 (12.45)	<0.001
>3 and ≤6	3,114 (20.43)	26.76 (15.94)		8.67 (8.16)		26.20 (12.46)	
>6 and ≤11	3,424 (22.46)	27.79 (15.80)		8.77 (8.18)		27.01 (12.19)	
>11	3,784 (24.82)	24.94 (16.09)		7.44 (7.62)		28.12 (12.86)	
**Contract status**							
Permanent	9,715 (63.75)	25.55 (15.91)	0.186	8.07 (7.83)	0.214	27.11 (12.55)	<0.001
Temporary	5,528 (36.27)	25.91 (15.96)		8.24 (7.89)		26.26 (12.47)	
**Professional title**							
Elementary or below	5,349 (35.09)	25.25 (16.08)	<0.001	8.31 (7.92)	<0.001	25.56 (12.57)	<0.001
Intermediate	5,861 (38.45)	27.71 (15.72)		8.78 (8.06)		26.90 (11.91)	
Senior	4,033 (26.46)	23.68 (15.77)		6.95 (7.29)		28.32 (13.15)	
**Ownership**							
Governmental	14,599 (95.78)	25.97 (15.96)	<0.001	8.20 (7.88)	<0.001	26.78 (12.50)	0.242
Non-governmental	644 (4.22)	21.51 (14.98)		6.51 (6.79)		27.37 (13.08)	
**Level of hospital**							
Three-grade level	10,152 (66.60)	26.14 (15.83)	<0.001	8.29 (7.87)	<0.001	26.86 (12.35)	0.513
Two-grade level	4,841 (31.76)	25.38 (16.14)		7.94 (7.84)		26.72 (12.81)	
Other	250 (1.64)	18.78 (14.95)		5.53 (6.53)		26.04 (14.07)	
**Shift work**							
Yes	13,288 (87.17)	26.76 (15.87)	<0.001	8.55 (7.98)	<0.001	26.50 (12.29)	<0.001
No	1,955 (12.83)	19.14 (14.81)		5.31 (6.19)		28.87 (13.87)	
**Workplace violence**							
Yes	13,699 (89.87)	27.13 (15.65)	<0.001	8.65 (7.92)	<0.001	26.95 (12.07)	<0.001
No	1,544 (10.13)	13.79 (13.27)		3.54 (5.27)		25.50 (15.97)	
**Self-perceived health status**							
Good	4,707 (30.88)	17.60 (14.04)	<0.001	5.54 (6.40)	<0.001	27.70 (14.25)	<0.001
Fair	7,729 (50.71)	26.85 (14.90)		8.31 (7.53)		26.53 (11.88)	
Poor	2,807 (18.42)	36.55 (14.38)		11.99 (9.14)		26.05 (11.01)	
**BMI (kg/m** ^**2**^ **)**							
<25	9,588 (62.90)	25.53 (15.86)	0.013	7.89 (7.67)	<0.001	26.64 (12.50)	0.032
≥25	5,655 (37.10)	26.20 (16.08)		8.54 (8.12)		27.09 (12.57)	
**History of hypertension**							
Yes	2,317 (15.20)	28.72 (16.21)	<0.001	9.38 (8.56)	<0.001	27.57 (12.16)	0.001
No	12,926 (84.80)	25.25 (15.84)		7.91 (7.69)		26.67 (12.59)	
**History of diabetes**							
Yes	598 (3.92)	28.19 (17.22)	<0.001	9.29 (9.01)	<0.001	27.15 (12.94)	0.496
No	14,645 (96.08)	25.68 (15.88)		8.08 (7.79)		26.79 (12.51)	
**History of coronary heart disease**							
Yes	471 (3.09)	34.17 (16.10)	<0.001	11.10 (9.29)	<0.001	28.33 (12.00)	0.007
No	14,772 (96.91)	25.51 (15.87)		8.04 (7.78)		26.75 (12.54)	
**Smoking status**							
Non-smokers	12,287 (80.61)	25.56 (15.88)	0.001	8.01 (7.73)	<0.001	26.64 (12.47)	0.001
Smokers	2,956 (19.39)	26.68 (16.16)		8.63 (8.32)		27.49 (12.73)	
**Alcohol drinking**							
Non-drinkers	1,1451 (75.12)	25.86 (15.90)	0.270	7.93 (7.71)	<0.001	26.78 (12.44)	0.753
Drinkers	3,792 (24.88)	25.53 (16.07)		8.74 (8.22)		26.86 (12.79)	
**Physical inactivity**							
Yes	11,098 (72.81)	27.29 (15.76)	<0.001	8.56 (7.94)	<0.001	26.49 (12.05)	<0.001
No	4,145 (27.19)	21.75 (15.73)		6.98 (7.49)		27.64 (13.69)	
**Sleeping quality**							
Good	2,295 (15.06)	15.39 (13.15)	<0.001	4.66 (5.74)	<0.001	28.55 (14.62)	<0.001
Fair	7,347 (48.20)	23.46 (14.91)		7.58 (7.31)		26.48 (12.63)	
Poor	5,601 (36.74)	33.09 (14.99)		10.28 (8.61)		26.51 (11.36)	
**CES-D scores**							
<20	9,818 (64.41)	18.54 (12.86)	<0.001	5.02 (5.36)	<0.001	27.40 (13.64)	<0.001
≥20	5,425 (35.59)	38.88 (12.18)		13.76 (8.47)		25.72 (10.12)	

*P-value is the result of ANOVA analysis*.

* *Associate's degree or vocational Diploma: Mainly cultivate vocational and technical students. Education level below bachelor's degree*.

*EE, Emotional Exhaustion; DP, Depersonalization; PA, Personal Accomplishment; BMI, Body Mass Index; CES-D, Center for EpidemiologicalStudies-Depression Scale*.

**Figure 1:**
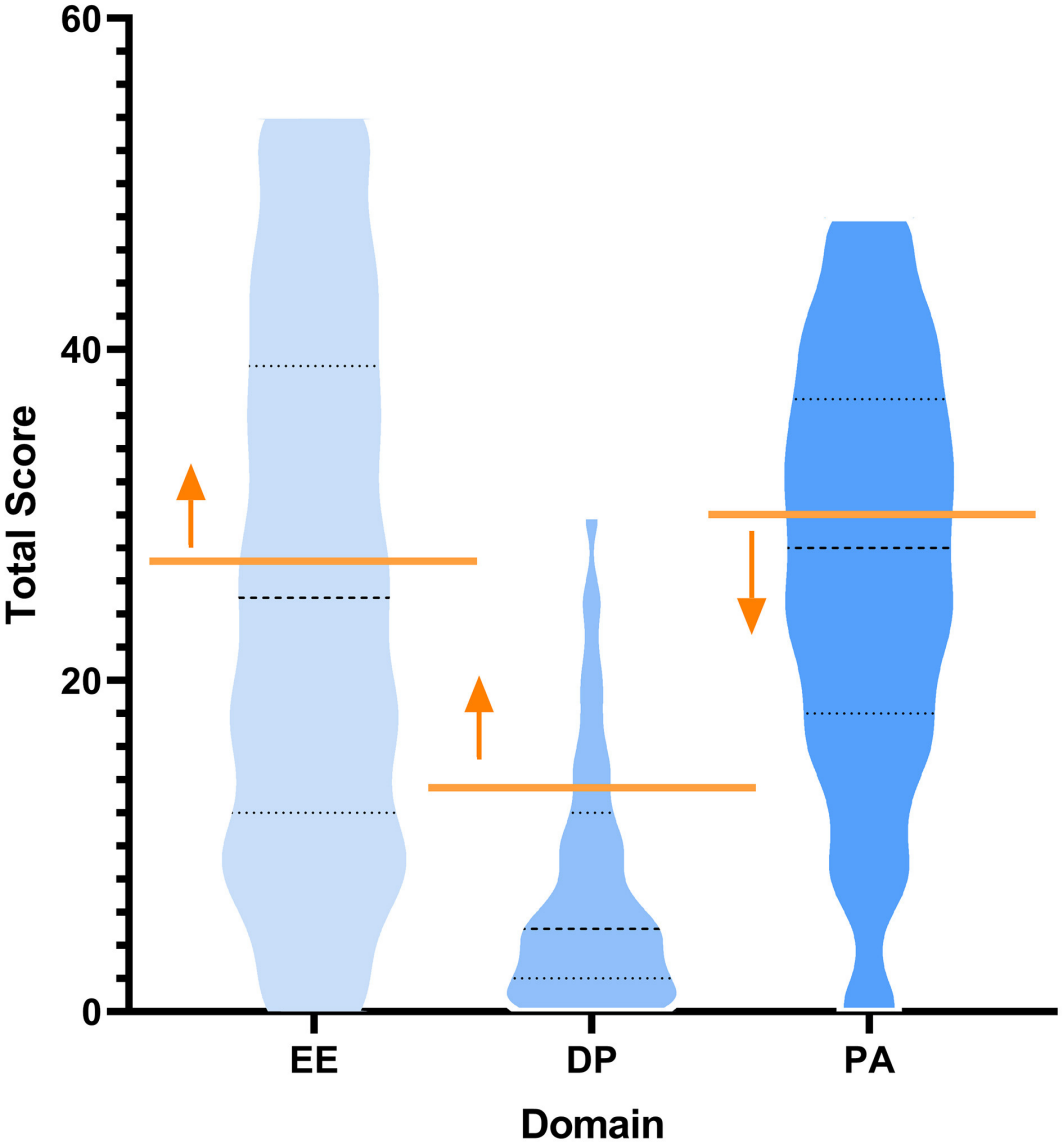
Violin plot showing threshold and distribution for burnout. Yellow lines indicate high burnout threshold with respect to each domain. Arrows indicate all participants who met or exceeded the high threshold for burnout. EE, emotional exhaustion; DP, depersonalization; PA, personal achievement.

The participants' mean scores were 25.8 (SD = 15.9; range = 0–54) on the EE subscale, 8.1 (SD = 7.9; range = 0–30) on the DP subscale, and 26.8 (SD = 12.5; range = 0–48) on the PA subscale, indicating a pattern of moderate EE, moderate DP, and high PA. The distribution of the sample is normally distributed. The plots of the mean domain score and thresholds can be seen in Figure 1. Table 2 shows the prevalence of burnout and its dimensions among emergency physicians. Among the participants, 46.8% reported high levels of EE burnout, 24.1% reported high levels on DP burnout, and 60.5% exhibited reduced feelings of PA. In total, 15.0% scored high for all three dimensions.

**Table 2:** The prevalence of burnout in emergency physicians according to three distinct dimensions of burnout.

**Variables**	**Burnout in all three dimensions**		**EE^a^**		**DP^b^**		**PA^c^**	
	***N***	**% (95%CI)**	***N***	**%**	***N***	**%**	***N***	**%**
Low^d^	1,301	8.5	5,098	33.4	8,601	56.4	3,112	20.4
Moderate	11,662	76.5	3,015	19.8	2,970	19.5	2,908	19.1
High^e^	2,280	15.0	7,130	46.8	3,672	24.1	9,223	60.5

*DP, Depersonalization; EE, Emotion exhaustion; PA, Personal accomplishment*.

a *Score ≤ 16 indicated low level; 17–26 indicated moderate level; ≥27 indicated high level*.

b *Score ≤ 6 indicated low level; 7–12 indicated moderate level; ≥13 indicated high level*.

c *Score ≥ 39 indicated low level; 32–38 indicated moderate level; ≤ 31 indicated high level*.

d *Low degree of burnout was defined by a low score on the subscales for EE and DP, and a high score on the PA subscale*.

e *High degree of burnout was defined by a high score on the subscales for EE and DP, and a low score on the PA subscale*.

The factors associated with the emergency physicians' burnout and three dimensions of burnout are presented in Table 3, including gender, age, region, education level, marital status, work tenure, professional title, ownership, shift work, workplace violence, self-perceived health status, BMI, history of hypertension and coronary heart disease, smoking and alcohol drinking status, physical inactivity, sleeping quality and depression.

**Table 3:** Correlates of burnout in emergency physicians: results of stepwise regression.

**Variables**	**EE**		**DP**		**PA**	
	**β**	**95%CI**	**β**	**95%CI**	**β**	**95%CI**
**Gender (Ref: male)**						
Female	1.776	1.334 to 2.217	−0.779	−1.023 to −0.535	1.995	1.527 to 2.462
**Age group, y (Ref: ≤31)**						
>31 and ≤37	—	—	—	—	—	—
>37 and ≤43	—	—	−0.401	−0.675 to −0.127	1.033	0.520 to 1.547
>43	—	—	−1.117	−1.411 to −0.823	2.551	2.025 to 3.077
**Region (Ref: less-developed)**						
Developed	0.598	0.206 to 0.990	0.332	0.117 to 0.548	0.709	0.243 to 1.175
Middle-developed	—	—	—	—	0.826	0.315 to 1.336
**Education level (Ref: associate's degree or vocational diploma)**						
Bachelor degree	2.411	1.601 to 3.221	1.089	0.640 to 1.539	—	—
Master degree or higher	2.827	1.954 to 3.701	1.446	0.960 to 1.932	—	—
**Marital status (Ref: married/widow/divorced)**						
Unmarried	—	—	0.361	0.069 to 0.654	—	—
**Income level (Ref: low)**						
High	—	—	—	—	—	—
Middle	—	—	—	—	—	—
**Work tenure, y (Ref: ≤3)**						
>3 and ≤6	—	—	—	—	—	—
>6 and ≤11	0.861	0.369 to 1.352	—	—	—	—
>11	0.600	0.120 to 1.080	—	—	—	—
**Contract status (Ref: temporary)**						
Permanent	—	—	—	—	—	—
**Professional title (Ref: elementary or below)**						
Intermediate	0.501	0.095 to 0.906	—	—	0.590	0.168 to 1.012
Senior	—	—	—	—	—	—
**Ownership (Ref: non-governmental)**						
Governmental	2.088	1.153 to 3.024	0.846	0.330 to 1.362	—	—
**Level of hospital (Ref: other)**						
Three-grade level	—	—	—	—	—	—
Two-grade level	—	—	—	—	—	—
**Shift work (Ref: no)**						
Yes	2.164	1.566 to 2.762	0.815	0.475 to 1.155	−0.965	−1.618 to −0.312
**Workplace violence (Ref: no)**						
Yes	5.802	5.160 to 6.444	2.152	1.798 to 2.506	2.406	1.730 to 3.082
**Self-perceived health status (Ref: good)**						
Fair	3.633	3.172 to 4.094	0.803	0.550 to 1.056	−0.933	−1.414 to −0.451
Poor	6.732	6.086 to 7.379	1.907	1.553 to 2.261	−1.185	−1.856 to −0.513
**BMI (kg/m** ^**2**^ **) (Ref: <25)**						
≥25	—	—	—	—	0.619	0.196 to 1.043
**History of hypertension (Ref: no)**						
Yes	0.748	0.196 to 1.299	0.478	0.169 to 0.788	—	—
**History of diabetes (Ref: no)**						
Yes	—	—	—	—	—	—
**History of coronary heart disease (Ref: no)**						
Yes	2.062	0.950 to 3.173	0.798	0.184 to 1.412	1.251	0.089 to 2.414
**Smoking status (Ref: non-smokers)**						
Smokers	0.761	0.259 to 1.264	—	—	1.397	0.869 to 1.925
**Alcohol drinking (Ref: non-drinkers)**						
Drinkers	—	—	0.742	0.485 to 0.999	—	—
**Physical inactivity (Ref: no)**						
Yes	1.168	0.727 to 1.609	—	—	—	—
**Sleeping quality (Ref: good)**						
Fair	2.898	2.321 to 3.475	0.905	0.588 to 1.222	−1.400	−2.005 to −0.794
Poor	5.757	5.103 to 6.410	0.819	0.460 to 1.178	−0.825	−1.509 to −0.141
**CES-D scores (Ref: <20)**						
≥20	16.137	15.707 to 16.568	7.663	7.426 to 7.901	−1.433	−1.886 to −0.980

*R^2^: EE: 0.457; DP: 0.318; PA: 0.025*.

*EE, Emotional Exhaustion; DP, Depersonalization; PA, Personal Accomplishment; BMI, Body Mass Index; CES-D, Center for Epidemiological Studies-Depression Scale*.

For emotional exhaustion, respondents who are female (β = 1.8) and smokers (β = 0.8), worked in developed country (β = 0.6) and governmental ownership (β = 2.1), had higher education level [bachelor degree (β = 2.4) or master degree or higher (β = 2.4)] and higher CES-D scores (β = 16.1), longer work tenure [>6 and ≤11 (β = 0.9) or >11 (β = 0.6)], intermediate professional title (β = 0.5), poorer self-perceived health status [fair (β = 3.6) or poor (β = 6.7)] and better sleeping quality [fair (β = 2.9) or poor (β = 5.8)], had history of hypertension (β = 0.7) and coronary heart disease (β = 2.1), participate in physical inactivity (β = 1.2), experienced shift work (β = 2.2), and workplace violence (β = 5.8) have higher EE scores.

For depersonalization, respondents who are unmarried (β = 0.4) and drinker (β = 0.7), worked in developed country (β = 0.3), had higher education level [bachelor degree (β = 1.1) or master degree or higher (β = 1.4)], poorer self-perceived health status [fair (β = 0.8) or poor (β = 2.0)] and poorer sleeping quality [fair (β = 0.9) or poor (β = 0.8)], had history of hypertension (β = 0.5), coronary heart disease (β = 0.8) and higher CES-D scores (β = 16.1) have higher DP scores; while those who are female (β = −0.8) and older [>37 and ≤43 (β = −0.4) or >43 (β = −1.1)] have lower DP scores.

For personal accomplishment, respondents who are female (β = 2.0), smoker (β = 1.4) and older [>37 and ≤43 (β = 1.0) or >43 (β = 2.6)], worked in middle-developed (β = 0.8) or developed country (β = 0.7), had intermediate professional title (β = 0.6), the BMI of over 25 (β = 0.6) and coronary heart disease (β = 1.3), experienced workplace violence (β = 2.4) have higher PA scores; while those who had poorer self-perceived health status [fair (β = −0.9) or poor (β = −1.2)], poorer sleeping quality [fair (β = −1.4) or poor (β = −0.8)] and higher CES-D scores (β = −1.4), experienced shift work (β = −1.0).

## Discussion

According to the findings of a previous meta-study (*N* = 4–7,830) (48), this study is the largest cross-sectional study of physician burnout in the world (7, 8). We found that 15.0% of the emergency physicians had a high level of burnout in China, with 46.9% scoring high for emotional exhaustion, 24.1% scoring high for depersonalization, and 60.5% having a high risk of low PA. Compared with a previous meta-analysis (17) on the level of burnout among emergency physicians (high EE = 40%, high DP = 41%, low PA = 35%), this study showed that more emergency physicians had high DP and low PA and fewer physicians had high DP. In addition, we identified a number of representative factors that clearly correlate with the development of occupational burnout among emergency physicians and reflect potential challenges in the practice of emergency physicians.

### Sleep Quality and Depression Were Significantly Associated With Burnout Among Emergency Physicians

Previous research has shown that job stress can have a variety of consequences, including damage to health (49), reduced quality of life (22), and psychological problems (24, 25). Another study reported that long-term work stress will lead to the potential for negative effects on the quality of patient care (50). This study demonstrates that these adverse effects ultimately lead to occupational burnout among emergency physicians. Because emergency physicians need to deal with patients in the acute stage, they are one of the groups experiencing the most stress among all types of physicians (27) and should be given full attention.

Studies have shown that chronic work stress can affect sleep quality, especially among physicians (22, 51). Emergency physicians are under great work pressure, and burnout occurs along with a decline in sleep quality. Similarly, the participants in this study with a previous history of hypertension and coronary heart disease showed higher emotional exhaustion and depersonalization. The results of a previous meta-study suggest that job stress has a significant impact on burnout (52). Therefore, emergency physicians must be alert to their high work burden and take corresponding measures to improve their quality of life.

Work stress can not only cause quality of life and health to decline but also take a toll on mental health (24, 53). Previous studies have suggested that a high workload can affect mental health (27, 54). This phenomenon is particularly common for emergency physicians and may lead to psychological problems such as depression, and depression is significantly correlated increased emotional exhaustion and depersonalization as well as a deficiency in personal accomplishment. Therefore, it is necessary to pay full attention to the mental health of emergency physicians and take targeted measures to improve their well-being, which is of great significance for maintaining the stability of emergency physician groups (24, 53).

### Emergency Physicians in Governmental Hospitals and Developed Areas Showed a High Level of Burnout

China's public hospitals, especially emergency departments, must receive a large number of patients (55). This is because Chinese residents generally trust governmental hospitals to maintain a high quality of medical care (especially in emergency situations); in addition, the Chinese government implements a universal health care policy, and the average cost of treatment in governmental hospitals is lower than that in non-governmental hospitals (56). Residents prefer to select governmental hospitals for medical treatment, and governmental hospitals face far more emergency demands than non-governmental hospitals, which leads to emergency physicians in governmental hospitals needing to treat a large number of emergency patients (55). In this study, as the workload increased, the emergency physicians' EE and DP increased. In recent years, the Chinese government has been working hard to develop the diagnosis and treatment capacity of non-governmental hospitals and reduce the cost of diagnosis and treatment to support non-governmental hospitals in sharing the excess demand of governmental hospitals (57).

In addition, emergency physicians in China also show different levels of burnout in different regions, with emergency physicians in developed regions having higher EE and DP.

This phenomenon may be the result of regional differences in the demand for medical services (29). Due to regional economic differences (32), the population of developed regions is much larger than that of less developed regions, so hospitals in developed regions face a greater burden, and the physicians in these hospitals are more prone to occupational burnout. In addition, highly skilled physicians are more likely to practice in developed regions, which further exacerbates the imbalance in the supply of health resources and induces more patients to seek medical treatment in developed regions (27). At present, the Chinese government has taken a series of measures to try to balance the supply of medical services in different regions, including through the development of remote diagnosis and treatment (58) and the construction of medical confederations to improve the level of diagnosis and treatment in underdeveloped areas and to reduce the workload of physicians in developed areas in the future.

### Physicians With Intermediate Titles and Those Who Worked Shift Work and Experienced Workplace Violence Were More Likely to Suffer From Burnout

One group that needs to be considered is physicians with intermediate titles. In previous studies, this group has not been given sufficient attention. However, this study found that physicians with intermediate professional titles had the highest EE and the highest PA, indicating that on the one hand, they had a higher sense of job achievement, but on the other hand, they had a higher sense of burnout. This finding is very interesting, and it is very likely that this phenomenon reflects the current state of physician practice in China. Compared with young physicians, physicians with intermediate titles are given more important tasks, and compared with physicians with senior professional titles, they need to work harder to gain promotion opportunities (59). Therefore, physicians with intermediate professional titles in China are the backbone of medical services and the hardest working physician group. However, due to the heavy work and family burdens that these physicians may face, they need to take care of their parents as well as their children and manage complex social relations, so they are more prone to burnout. More attention should be given to the physical and mental health statuses of middle-aged physicians, which should be the focus of health researchers and policy makers.

Another group that needs to be considered is emergency physicians who engage in shift work, who have higher EE and DP as well as significantly lower PA than those who do not. Previous studies have documented the physical effects of shift work, which can lead to decreased energy levels and physical and mental health problems (36, 60). Emergency physicians are required to provide emergency medical services around the clock, so most emergency physicians have to perform shift work, which is especially common in China, where a large amount of medical services must be provided. Therefore, the managers of medical institutions should further improve the scientific basis of management (61) and avoid emergency physicians having to perform shift work.

Physician-patient conflict also affects the occupational burnout of emergency physicians. In recent years, conflict between physicians and patients has become increasingly prominent in China (37). This conflict mainly refers to the imbalance between the supply and demand of medical services but also reflects the tension in interpersonal relations, the frequent occurrence of violent incidents and the instability of the social environment (38). In the face of workplace violence, physicians may become suspicious of medical services, which in turn reduces the quality of medical services provided and ultimately damages patients' health (39). Physicians are also more prone to burnout and occupational rejection after being exposed to violence. This study proves that workplace violence has a strong impact on occupational burnout, which leads to an increase in EE and DP and a decrease in PA. In recent years, China has taken an increasing number of measures to improve the relationship between physicians and patients and maintain the professional image of physicians, which will help to maintain the stability of the emergency physician group (27).

Organizational psychology research has explored ways to ameliorate physician burnout. Gagné and Deci suggested that financial incentives may not directly prevent or reduce burnout (60), and our results support the hypothesis that income level may not be a factor in burnout among emergency physicians. In reality, Gagné and Deci posited three pillars that support professionals' intrinsic motivation and psychological well-being: competence, autonomy, and relatedness (60). However, in China, emergency physicians with only intermediate professional titles have to take on a large amount of work, which is not in line with the principle of competence. Management systems that are not based on scientific evidence rob emergency physicians of the right to take volitional action and force them to perform shift work and comply with other unreasonable institutional arrangements. In addition, the high incidence of workplace violence undermines the intimacy of the doctor-patient relationship. Our results support previous suggestions that stripping away these three competencies may be a deeper cause of physician burnout. Therefore, an important direction for future research is to explore strategies to reduce physicians' burnout from the perspective of organizational psychology.

Finally, this study found that several demographic characteristics also influenced the burnout of emergency physicians to varying degrees. Studies have shown that female physicians have higher EE and PA, while male physicians have higher DP, and that burnout declines as physicians age. In addition, the physicians in this study who had higher education levels, who were unmarried, and who had been in the emergency department for longer were more likely to report burnout. In conclusion, this study systematically demonstrated the potential sources of occupational burnout among emergency physicians by analyzing various factors. Together with previous evidence that burnout can effectively be reduced with moderate levels of investment, these findings suggest substantial economic value of policy and organizational expenditures for burnout reduction programs for physicians (6). Therefore, appropriate measures should be taken to reduce the job burnout of emergency physicians according to the findings of this study.

### Strengths and Limitations

Conducted in China, the largest developing country in the world, our study used a large-sample cross-sectional survey to explore the influencing factors of occupational burnout among emergency physicians. First, the large sample size significantly increased the statistical power to detect burnout. We surveyed nearly a quarter (19) of the nation's emergency physicians, so the study was representative. Second, this study presented a broad view of the challenges faced by emergency physicians and reflects the practice status of physicians in China. This study closely examined the challenges faced by emergency physicians, such as decreased sleep quality, poor physical condition, depression, and workplace violence, and demonstrated the impact of these factors on burnout. Through quantitative analysis, it was found that working in public hospitals, working in developed areas and performing shift work were important reasons for increased occupational burnout in emergency physicians. In particular, the study innovatively proposed that emergency physicians with intermediate titles have the highest sense of achievement and burnout, which provides a reference for future research. Third, the survey was anonymous and self-administered, which likely made the respondents provide more valid responses and eliminated interviewer bias.

This study had several limitations. First, the study used a cross-sectional study design, which precluded the evaluation of the temporality of the observed relationships. Second, the data were collected from the participants' self-reports; thus, recall bias was unavoidable. Finally, the study was performed before the COVID-19 pandemic, which may have led to changes in the burnout of emergency physicians after the pandemic.

### Suggestions for Future Research

The findings of our study can serve as a reference for further research in the future. Based on our findings, we suggest that, first, prospective studies are needed to investigate the association between the identified factors and burnout. Second, this study highlights the need for the investigation or implementation of interventions to improve emergency physicians' well-being or promote strategies to reduce work burnout on emergency physicians in China's emergency healthcare settings, include more rest time, more flexible schedules, and adequate salary. Finally, investigating the potential impact of burnout on emergency physicians' work performance, the quality of patient care delivery, and family life would provide important insights.

## Conclusion

The results of the large-sample survey showed that 14.9% of emergency physicians had a high level of burnout in China, with 46.78% scoring high for EE, 24.1% scoring high for DP, and 60.5% having a high risk of low PA. Self-perceived health status, sleep quality, depression, region, ownership, professional title, workplace violence, shift work and other factors were the main factors associated with the prevalence of burnout. Positive measures should be taken to reduce the burnout of emergency physicians and improve their work enthusiasm to maintain the quality of emergency medical services.

## Data Availability Statement

The raw data supporting the conclusions of this article will be made available by the authors, without undue reservation.

## Ethics Statement

The studies involving human participants were reviewed and approved by the Institutional Ethics Board of the Second Affiliated Hospital of Hainan Medical University, Haikou, China, in accordance with the Chinese Statistical Law. The patients/participants provided their written informed consent to participate in this study.

## Author Contributions

SY, XS, YG, and CL conceived and designed the study. RW and ZL participated in the acquisition of data. XS analyzed the data. SY gave advice on methodology. SY and XS drafted the manuscript. XH, YG, and CL revised the manuscript. CL is the guarantors of this work and had full access to all the data in the study and takes responsibility for its integrity and the accuracy of the data analysis. All authors read and approved the final manuscript.

## Funding

This study was supported by the National Natural Science Foundation of China (82160647) and the Hainan Provincial Key Research and Development Project (ZDYF2020112).

## Conflict of Interest

The authors declare that the research was conducted in the absence of any commercial or financial relationships that could be construed as a potential conflict of interest.

## Publisher's Note

All claims expressed in this article are solely those of the authors and do not necessarily represent those of their affiliated organizations, or those of the publisher, the editors and the reviewers. Any product that may be evaluated in this article, or claim that may be made by its manufacturer, is not guaranteed or endorsed by the publisher.

## Abbreviations

EE: Emotional Exhaustion
DP: Depersonalization
PA: Personal Accomplishment
BMI: Body Mass Index
CES-D: Center for Epidemiological Studies-Depression Scale.

## References

[1] Jin MU Jeong SH Kim EK Choi YH Song KB. Burnout and its related factors in Korean dentists. Int Dent J. (2015) 65:22–31. 10.1111/idj.12142 25412905 PMC9376508

[2] Maslach C Schaufeli WB Leiter MP. Job burnout. Annu Rev Psychol. (2001) 52:397–422. 10.1146/annurev.psych.52.1.397 11148311

[3] Panagioti M Geraghty K Johnson J. How to prevent burnout in cardiologists? A review of the current evidence, gaps, and future directions. Trends Cardiovasc Med. (2018) 28:1–7. 10.1016/j.tcm.2017.06.018 28716506

[4] Aiken LH Clarke SP Sloane DM Sochalski J Silber JH. Hospital nurse staffing and patient mortality, nurse burnout, and job dissatisfaction. JAMA. (2002) 288:1987–93. 10.1001/jama.288.16.1987 12387650

[5] Adriaenssens J De Gucht V Maes S. Determinants and prevalence of burnout in emergency nurses: a systematic review of 25 years of research. Int J Nurs Stud. (2015) 52:649–61. 10.1016/j.ijnurstu.2014.11.004 25468279

[6] Han S Shanafelt TD Sinsky CA Awad KM Dyrbye LN Fiscus LC. Estimating the attributable cost of physician burnout in the United States. Ann Intern Med. (2019) 170:784–90. 10.7326/M18-1422 31132791

[7] Lepnurm R Lockhart WS Keegan D. A measure of daily distress in practising medicine. Can J Psychiatry. (2009) 54:170–80. 10.1177/070674370905400305 19321021

[8] Shanafelt TD Boone S Tan L Dyrbye LN Sotile W Satele D. Burnout and satisfaction with work-life balance among US physicians relative to the general US population. Arch Intern Med. (2012) 172:1377–85. 10.1001/archinternmed.2012.3199 22911330

[9] Gulalp B Karcioglu O Sari A Koseoglu Z. Burnout: need help? J Occup Med Toxicol. (2008) 3:32. 10.1186/1745-6673-3-32 19061497 PMC2621227

[10] Arora M Asha S Chinnappa J Diwan AD. Review article: burnout in emergency medicine physicians. Emerg Med Australas. (2013) 25:491–5. 10.1111/1742-6723.12135 24118838

[11] Xu T Xu J Yu X Ma S Wang Z. Clinical decision-making by the emergency department resident physicians for critically ill patients. Front Med-Prc. (2012) 6:89–93. 10.1007/s11684-012-0183-9 22460453

[12] Lu X Xu S. Important role of emergency department doctors after the outbreak of COVID-19 in China. Br Assoc Accident Emerg Med. (2020) 37:334. 10.1136/emermed-2020-209633 32366617

[13] Durand AC Bompard C Sportiello J Michelet P Gentile S. Stress and burnout among professionals working in the emergency department in a French university hospital: prevalence and associated factors. Work. (2019) 63:57–67. 10.3233/WOR-192908 31127745

[14] Moukarzel A Michelet P Durand AC Sebbane M Bourgeois S Markarian T. Burnout syndrome among emergency department staff: prevalence and associated factors. Biomed Res Int. (2019) 2019:6462472. 10.1155/2019/6462472 30800675 PMC6360614

[15] Lu DW Weygandt PL Pinchbeck C Strout TD. Emergency medicine trainee burnout is associated with lower patients' satisfaction with their emergency department care. AEM Educ Train. (2018) 2:86–90. 10.1002/aet2.10094 30051074 PMC6001511

[16] Baier N Roth K Felgner S Henschke C. Burnout and safety outcomes - a cross-sectional nationwide survey of EMS-workers in Germany. BMC Emerg Med. (2018) 18:24. 10.1186/s12873-018-0177-2 30126358 PMC6102842

[17] Zhang Q Mu M He Y Cai Z Li Z. Burnout in emergency medicine physicians: a meta-analysis and systematic review. Medicine. (2020) 99:e21462. 10.1097/MD.0000000000021462 32769876 PMC7593073

[18] The Lancet. Physician burnout: a global crisis. Lancet. (2019) 394:93. 10.1016/S0140-6736(19)31573-9 31305255

[19] Pan C Pang J Cheng K Xu F Chen Y. Trends and challenges of emergency and acute care in Chinese mainland: 2005-2017. World J Emerg Med. (2021) 12:5–11. 10.5847/wjem.j.1920-8642.2021.01.001 33505543 PMC7790704

[20] Zhou H Li C Yan Y. The emergency department in China: status and challenges. Br Assoc Accident Emerg Med. (2014) 31:85–6. 10.1136/emermed-2013-202912 23811855

[21] Williams ES Konrad TR Scheckler WE Pathman DE Linzer M McMurray JE. Understanding physicians' intentions to withdraw from practice: the role of job satisfaction, job stress, mental and physical health. (2001). Health Care Manage Rev. (2010) 35:105–15. 10.1097/01.HMR.0000304509.58297.6f 20234217

[22] Sun T Gao L Li F Shi Y Xie F Wang J. Workplace violence, psychological stress, sleep quality and subjective health in Chinese doctors: a large cross-sectional study. BMJ Open. (2017) 7:e17182. 10.1136/bmjopen-2017-017182 29222134 PMC5728267

[23] Yang G Li C Zhu X Yan J Liu J. Prevalence of and risk factors associated with sleep disturbances among HPCD exposed to COVID-19 in China. Sleep Med. (2021) 80:16–22. 10.1016/j.sleep.2020.12.034 33540240 PMC7834103

[24] Song X Fu W Liu X Luo Z Wang R Zhou N. Mental health status of medical staff in emergency departments during the Coronavirus disease 2019 epidemic in China. Brain Behav Immun. (2020) 88:60–5. 10.1016/j.bbi.2020.06.002 32512134 PMC7273140

[25] Kang L Ma S Chen M Yang J Wang Y Li R. Impact on mental health and perceptions of psychological care among medical and nursing staff in Wuhan during the 2019 novel coronavirus disease outbreak: a cross-sectional study. Brain Behav Immun. (2020) 87:11–7. 10.1016/j.bbi.2020.03.028 32240764 PMC7118532

[26] Fu C Wang G Shi X Cao F. Social support and depressive symptoms among physicians in tertiary hospitals in China: a cross-sectional study. BMC Psychiatry. (2021) 21:217. 10.1186/s12888-021-03219-w 33926402 PMC8082214

[27] Hu Y Zhang Z. Skilled doctors in tertiary hospitals are already overworked in China. The Lancet Global Health. (2015) 3:e737. 10.1016/S2214-109X(15)00192-8 26566744

[28] Chen J Xu J Zhang C Fu X. Medical professionalism among clinical physicians in two tertiary hospitals, China. Soc Sci Med 1982. (2013) 96:290–6. 10.1016/j.socscimed.2012.09.044 23102754

[29] Yu X Zhang W. All-cause mortality rate in China: do residents in economically developed regions have better health? Int J Equity Health. (2020) 19:12. 10.1186/s12939-020-1128-6 31964379 PMC6975071

[30] Li J Liu J Ma Y Peng P He X Guo W. Imbalanced regional development of acute ischemic stroke care in emergency departments in China. Emerg Med Int. (2019) 2019:3747910. 10.1155/2019/3747910 31467718 PMC6701302

[31] Zhou L Xu X Antwi HA Wang L. Towards an equitable healthcare in China: evaluating the productive efficiency of community health centers in Jiangsu Province. Int J Equity Health. (2017) 16:89. 10.1186/s12939-017-0586-y 28545456 PMC5445518

[32] Nong S Yao NA. Reasons behind stymied public hospital governance reform in China. PLoS ONE. (2019) 14:e222204. 10.1371/journal.pone.0222204 31498814 PMC6733505

[33] Zhao D Zhang Z. Qualitative analysis of direction of public hospital reforms in China. Front Med. (2018) 12:218–23. 10.1007/s11684-017-0534-7 28674836

[34] Yip WC Hsiao WC Chen W Hu S Ma J Maynard A. Early appraisal of China's huge and complex health-care reforms. Lancet. (2012) 379:833–42. 10.1016/S0140-6736(11)61880-1 22386036

[35] Kimo TJ Ramoska EA Clark TR Hansoti B Dougherty J Freeman W. Factors associated with burnout during emergency medicine residency. Acad Emerg Med. (2014) 21:1031–5. 10.1111/acem.12464 25269584

[36] Angerer P Schmook R Elfantel I Li J. Night work and the risk of depression. DTSCH Arztebl Int. (2017) 114:404–11. 10.3238/arztebl.2017.0404 28669378 PMC5499504

[37] Wu Y Jiang F Ma J Tang Y Wang M Liu Y. Experience of medical disputes, medical disturbances, verbal and physical violence, and burnout among physicians in China. Front Psychol. (2021) 11:556517. 10.3389/fpsyg.2020.556517 33584400 PMC7878671

[38] Xu W. Violence against doctors in China. Lancet. (2014) 384:745. 10.1016/S0140-6736(14)61438-0 25176548

[39] Panagioti M Geraghty K Johnson J Zhou A Panagopoulou E Chew-Graham C. Association between physician burnout and patient safety, professionalism, and patient satisfaction: a systematic review and meta-analysis. JAMA Intern Med. (2018) 178:1317–31. 10.1001/jamainternmed.2018.3713 30193239 PMC6233757

[40] Maslach C Jackson SE. The measurement of experienced burnout. J Organ Behav. (1981) 2:99–113. 10.1002/job.403002020525855820

[41] Maslach C. JSLM Maslach Burnout Inventory. Menlo Park, CA: Mind Garden Inc. (1996).

[42] Cosco TD Lachance CC Blodgett JM Stubbs B Co M Veronese N. Latent structure of the centre for epidemiologic studies depression scale (CES-D) in older adult populations: a systematic review. Aging Ment Health. (2020) 24:700–4. 10.1080/13607863.2019.1566434 30661386

[43] Dozeman E van Schaik DJ van Marwijk HW Stek ML van der Horst HE Beekman AT. The center for epidemiological studies depression scale (CES-D) is an adequate screening instrument for depressive and anxiety disorders in a very old population living in residential homes. Int J Geriatr Psychiatry. (2011) 26:239–46. 10.1002/gps.2519 20623777

[44] Cheng ST Chan AC. The center for epidemiologic studies depression scale in older Chinese: thresholds for long and short forms. Int J Geriatr Psychiatry. (2005) 20:465–70. 10.1002/gps.1314 15852439

[45] Vilagut G Forero CG Barbaglia G Alonso J. Screening for depression in the general population with the center for epidemiologic studies depression (CES-D): a systematic review with meta-analysis. PLoS ONE. (2016) 11:e155431. 10.1371/journal.pone.0155431 27182821 PMC4868329

[46] Jiang L Wang Y Zhang Y Li R Wu H Li C. The reliability and validity of the center for epidemiologic studies depression scale (CES-D) for Chinese university students. Front Psychiatry. (2019) 10:315. 10.3389/fpsyt.2019.00315 31178764 PMC6537885

[47] Wang PX Wang MZ Hu GX Wang ZM. Study on the relationship between workplace violence and work ability among health care professionals in Shangqiu City. Wei Sheng Yan Jiu. (2006) 35:472–4. 16986527

[48] Rotenstein LS Torre M Ramos MA Rosales RC Guille C Sen S. Prevalence of burnout among physicians: a systematic review. JAMA. (2018) 320:1131–50. 10.1001/jama.2018.12777 30326495 PMC6233645

[49] Hersch RK Cook RF Deitz DK Kaplan S Hughes D Friesen MA. Reducing nurses' stress: a randomized controlled trial of a web-based stress management program for nurses. Appl Nurs Res. (2016) 32:18–25. 10.1016/j.apnr.2016.04.003 27969025 PMC5159423

[50] Schrijver I. Pathology in the medical profession? Taking the pulse of physician wellness and burnout. Arch Pathol Lab Med. (2016) 140:976–82. 10.5858/arpa.2015-0524-RA 26828114

[51] Xiao Y Wang J Chen S Wu Z Cai J Weng Z. Psychological distress, burnout level and job satisfaction in emergency medicine: a cross-sectional study of physicians in China. Emerg Med Australas. (2014) 26:538–42. 10.1111/1742-6723.12315 25319720

[52] Watanabe M Yamauchi K. The effect of quality of overtime work on nurses' mental health and work engagement. J Nurs Manag. (2018) 26:679–88. 10.1111/jonm.12595 29682824

[53] Maharaj S Lees T Lal S. Prevalence and risk factors of depression, anxiety, and stress in a cohort of Australian nurses. Int J Environ Res Public Health. (2018) 16:61. 10.3390/ijerph16010061 30591627 PMC6339147

[54] Ni T Chen M Zhou W Zhao J Jia D. Difference of achievements between physicians from public hospitals and emergency Medical Center in prehospital emergency. Medicine. (2018) 97:e13070. 10.1097/MD.0000000000013070 30383688 PMC6221651

[55] Yip W Hsiao W. Harnessing the privatisation of China's fragmented health-care delivery. Lancet. (2014) 384:805–18. 10.1016/S0140-6736(14)61120-X 25176551 PMC7159287

[56] Han Y Lie RK Guo R. The internet hospital as a Telehealth model in China: systematic search and content analysis. J Med Internet Res. (2020) 22:e17995. 10.2196/17995 32723721 PMC7424477

[57] Yip WC Hsiao W Meng Q Chen W Sun X. Realignment of incentives for health-care providers in China. Lancet. (2010) 375:1120–30. 10.1016/S0140-6736(10)60063-3 20346818

[58] Maltese F Adda M Bablon A Hraeich S Guervilly C Lehingue S. Night shift decreases cognitive performance of ICU physicians. Intens Care Med. (2016) 42:393–400. 10.1007/s00134-015-4115-4 26556616

[59] Panagioti M Panagopoulou E Bower P Lewith G Kontopantelis E Chew-Graham C. Controlled interventions to reduce burnout in physicians: a systematic review and meta-analysis. JAMA Intern Med. (2017) 177:195–205. 10.1001/jamainternmed.2016.7674 27918798

[60] Gagné M Deci EL. Self-determination theory and work motivation. J Organ Behav. (2005) 26:331–62. 10.1002/job.32225855820

[61] Friedberg MW Chen PG Van Busum KR Aunon F Pham C Caloyeras J. Factors affecting physician professional satisfaction and their implications for patient care, health systems, and health policy. Rand Health Q. (2014) 3:1. 10.7249/RB9740 28083306 PMC5051918

